# Immunophenotyping in Myelodysplastic Syndromes Can Add Prognostic Information to Well-Established and New Clinical Scores

**DOI:** 10.1371/journal.pone.0081048

**Published:** 2013-12-06

**Authors:** Suiellen C. Reis-Alves, Fabíola Traina, Guilherme Harada, Paula M. Campos, Sara T. O. Saad, Konradin Metze, Irene Lorand-Metze

**Affiliations:** 1 Hematology and Hemotherapy Center, University of Campinas - Campinas, Sao Paulo, Brazil; 2 Faculty of Medicine, University of Campinas – Campinas, Sao Paulo, Brazil; Cardiff University, United Kingdom

## Abstract

**Background:**

myelodysplastic syndromes (MDS) are a heterogeneous group of hematopoietic clonal disorders. So, prognostic variables are important to separate patients with a similar biology and clinical outcome. We compared the importance of risk stratification in primary MDS of IPSS and WPSS with the just described revision of IPSS (IPSS-R), and examined if variables obtained by bone marrow immunophenotyping could add prognostic information to any of the scores.

**Methods:**

In this prospective study of 101 cases of primary MDS we compared the relation of patients’ overall survival with WHO types, IPSS, IPSS-R, WPSS and phenotypic abnormalities of hematopoietic precursors. We examined aberrancies in myelomonocytic precursors and CD34^+^ cells. Patients were censored when receiving chemotherapy or BM transplantation. Survival analysis was made by Cox regressions and stability of the models was examined by bootstrap resampling.

**Results:**

median age: 64 years (15–93). WHO types: 2 cases of 5q- syndrome, 7 of RA, 64 of RCDM and 28 of RAEB. In the univariate Cox analysis, increasing risk category of all scores, degree of anemia, higher percentage of BM blasts, higher number of CD34^+^ cells and their myeloid fractions besides increasing number of phenotypic abnormalities detected were significantly associated with a shorter survival. In the multivariate analysis comparing the three scores, IPSS-R was the only independent risk factor. Comparing WPSS with phenotypic variables (CD34^+^/CD13^+^ cells, CD34^+^/CD13^−^ cells and “total alterations”) the score and “CD34^+^/CD13^+^ cells” remained in the model. When IPSS was tested together with these phenotypic variables, only “CD34^+^/CD13^+^ cells”, and “total alterations” remained in the model. Testing IPSS-R with the phenotypic variables studied, only the score and “CD34^+^/CD13^+^ cells” entered the model.

**Conclusions:**

Immunophenotypic analysis of myelomonocytic progenitors provides additional prognostic information to all clinical scores studied. IPSS-R improved risk stratification in MDS compared to the former scores.

## Introduction

Myelodysplastic Syndromes (MDS) constitute a wide spectrum of hematopoietic clonal disorders with a variable clinical course [1]–[5]. In this context, the study of features that are able to predict patients’ survival and progression to AML is very important [4]–[10], in order to assign patients correctly in clinical trials which test new treatment approaches.

The severity of PB cytopenias, the percentage of BM blasts and the kind of cytogenetic abnormalities found have long been recognized as independent prognostic factors in MDS, and have been included in the currently used prognostic scores, such as IPSS and WPSS [4]–[10].

The IPSS, [6] that is currently the most frequently used risk stratification score for MDS, is based on the number of cytopenias found in peripheral blood counts (PB), percentage of bone marrow (BM) blasts and kind of cytogenetic abnormalities. This score has recently been revised (IPSS-R) [9] as the importance of karyotype abnormalities was underscored in the former classification system. Cytogenetic findings were re-analyzed in a large international multicentric study and their importance for risk stratification was revised [7]. Besides, IPSS-R risk categories are based not only on these revised cytogenetic groups, but also on a more detailed categorization of the peripheral blood values and BM blast counts [9]. Therefore, a considerable proportion of cases with IPSS intermediate risk switched to a higher risk category.

Karyotype abnormalities are found in 30%–80% of cases with primary MDS. They are very heterogeneous, and some abnormalities are very rare, precluding the assessment of their real prognostic value [8]. On the other hand, several frequent point mutations such as TP53, EHZ2, ETV6, RUNX1 and ASXL1 have been considered independent prognostic factors when compared to age, sex and IPSS classification [11], [12]. TET2 mutations were frequent in cases with a normal karyotype and those of TP53 were associated with abnormalities of chromosome 17 or a complex karyotype, but mutations of EZH2 (localized in chromosome 7) were not associated with 7q deletion. So, the presence of multiple gene mutations in MDS may help to explain the clinical heterogeneity of these clonal disorders and could help to improve the prediction of the patients’ prognosis.

In 2005, a WHO classification-based Prognostic Scoring System (WPSS), considering WHO categories, transfusion requirement, and karyotype abnormalities (risk categories as in IPSS) was described. As transfusion dependency is difficult to standardize, this parameter was substituted by the hemoglobin level of the patient: <9 g/dL for males and <8 g/dL for females [10]. This score is dynamic, and can be applied at every point in the course of the disease.

In the last decade, multiparametric flow cytometric analysis of BM hematopoietic precursors has been extensively studied in MDS and is nowadays recognized as a useful diagnostic tool, especially in cases with a normal karyotype [13]–[22]. Immunophenotyping in MDS is based on the knowledge that antigen expression during maturation of normal hematopoiesis is tightly controlled. In MDS, deviations of the normal pattern, with over- or underexpression of antigens, as well as maturation asynchrony are indicative of a clonal abnormality [13]–[22]. Increased number and aberrant antigen expression of CD34^+^ cells, as well as the total number of phenotypic abnormalities in BM precursors, [13], [14], [17]–[22] have shown to be independent risk factors for survival.

Immunophenotyping in MDS is feasible in all patients, nowadays reasonably well standardized, and has been recommended as an ancillary diagnostic tool for the differential diagnosis of MDS with a normal karyotype and non-clonal reactive PB cytopenias [13]–[16], [18], [23].

So, many independent prognostic variables and risk stratification scores have been described in MDS. However, a head-to-head comparison of them has seldom been performed. Especially it is not clear at the moment, to which degree the IPSS-R increases the prognostic power when compared with the old IPSS classification. Therefore, the aim of our prospective study was to compare in patients with primary MDS the two well-established scores (WPSS and IPSS) with the newly created IPSS-R classification and to investigate whether variables obtained by BM flow cytometric analysis could add prognostic information to any of these scores.

## Methods

### Patients

We analyzed prospectively 87 cases with adult primary MDS diagnosed at our Institution between 2006 and 2012 and 14 patients who had already been diagnosed at other Institutions and were referred to us, but had not received any treatment. The project was approved by the Ethics Committee of the Faculty of Medical Sciences, University of Campinas (Proc 0652.0.146.000-08).

Diagnosis was based on clinical data, PB counts, BM cytology and histology, as well as cytogenetics, as recommended by the WHO criteria. Stability of PB counts for at least 3 months was required for all cases. Non-clonal disorders presenting PB cytopenias and a cellular marrow with hematopoietic cell atypias (HIV, hepatitis and other viral infections, autoimmune disorders, etc) were excluded. At the time of the patients’ evaluation none of them was being treated.

For cytogenetic examination, nucleated cells from aspirated bone marrow were separated by Ficoll-Hypaque centrifugation and then cultured for 48 hours in RPMI1640 supplemented with calf fetal serum without stimulation. Finally, G-banding was performed. Results were reported using the ISCN (International Standing Committee on Human Cytogenetic Nomenclature) criteria [24].

The cases were classified by WHO criteria and the risk was accessed according to IPSS, IPSS-R and WPSS using the hemoglobin level instead of “transfusion dependency” [10]. Only patients with a high risk disease according to WHO criteria (refractory anemia with excess of blasts - RAEB) were considered for specific treatment with cytotoxic therapy. The other patients received only supportive care.

All PB and BM samples for the study were obtained from each patient after written informed consent.

### Flow Cytometric Analysis

Flow cytometric analysis was performed as previously described [15], [17], [20]. In brief, aspirated BM was collected in EDTA and diluted to obtain a concentration of 5–7×10^6^ cells in 100 µl per test. Immunofluorescence staining was made using a standardized direct lyse-and-wash technique within 24 hours after sample collection following the European LeukemiaNet (ELN) recommendations [15], [23]. Antigenic expression of the myelomonocytic series and CD34^+^ cell subsets were analyzed using four color combinations of MoAbs: CD64/CD14/CD45/HLA-DR; CD16/CD11b/CD45/CD13; CD13/CD117/CD45/CD34; CD19/CD10/CD45/CD34 and CD7/CD56/CD45/CD34 (Table 1).

**Table 1:** Antibodies used in the panel, with clones and sources.

Monoclonalantibodies	Fluorochromes	Clone	Source
CD13	APC	WM-15	Pharmingen
CD13	FITC	WM-47	Dako
CD14	PE	RMO52	Beckman Coulter
CD16	FITC	3G8	Pharmingen
CD11b	PE	2LPM19c	Dako
CD19	FITC	HD37	Dako
CD64	FITC	10.1	eBioscience
CD7	FITC	DK24	Dako
CD56	PE	MeM-188	ExBio
CD34	APC	8G12	Becton Dickinson
CD45	PerCP	2D1	Becton Dickinson
CD117	PE	104D2	Dako
HLA-DR	FITC	AB3	Dako

**Figure 1:**
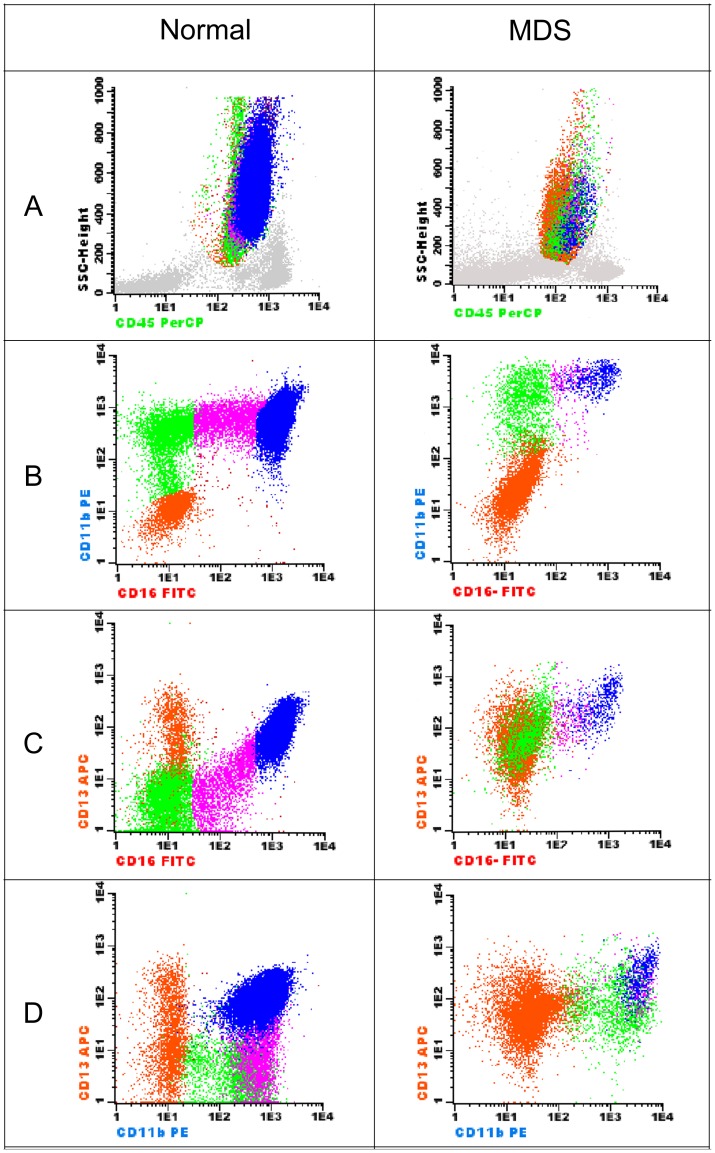
Detection of abnormalities in the granulocytic maturation: plots found in a normal BM on the left and those in a case of RCMD on the right (combination: CD16/CD11b/CD45/CD13). The granulocytic population was selected on the SSC/CD45 plot (A). The four differentiation stages are distinguishable based on the expression of CD11b, CD16 and CD13 (B–D: orange: promyelocytes; green: myelocytes; purple: metamyelocytes and blue: mature neutrophils). Maturing granulocytes show an abnormal decreased granularity demonstrated by a low SSC (A). Increase of a uniform population with the phenotype CD11b^low^/CD13^low^/CD16^−^ (promyelocytes) with a gap of maturation between them and myelocytes. Abnormally high expression of CD13 in myelocytes and metamyelocytes (C and D).

This platform is able to reveal up to 16 alterations in the granulocytic and monocytic cell lines as well as in CD34^+^ progenitors [14], [15], [17], [20], [23]:

Granulocytic precursors (4 abnormalities): decreased SSC, abnormal maturation pattern in the CD16/CD13 and CD16/CD11b combinations (deficiency or increase in antigen expression or asynchronous maturation) and expression of CD34 in >10% of mature granulocytes (Fig. 1).

**Figure 2:**
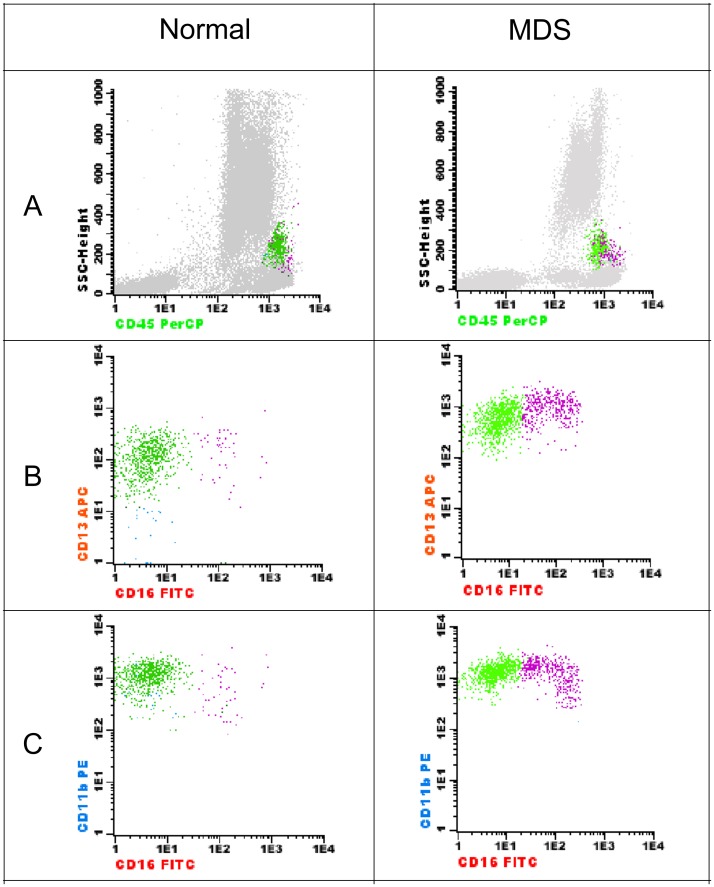
Detection of abnormalities in monocytes in the combination CD16/CD11b/CD45/CD13. Left: normal BM. Right: a case of RCMD (monocytes = 2.4%). Abnormal continuous acquisition of CD16 in the mature monocytes (B).

Monocytic cell line (4 abnormalities): increased number, increase in CD16^+^ monocytes, asynchronous/abnormal maturation pattern and expression of CD34 in >10% of the cells (Fig. 2).

**Figure 3:**
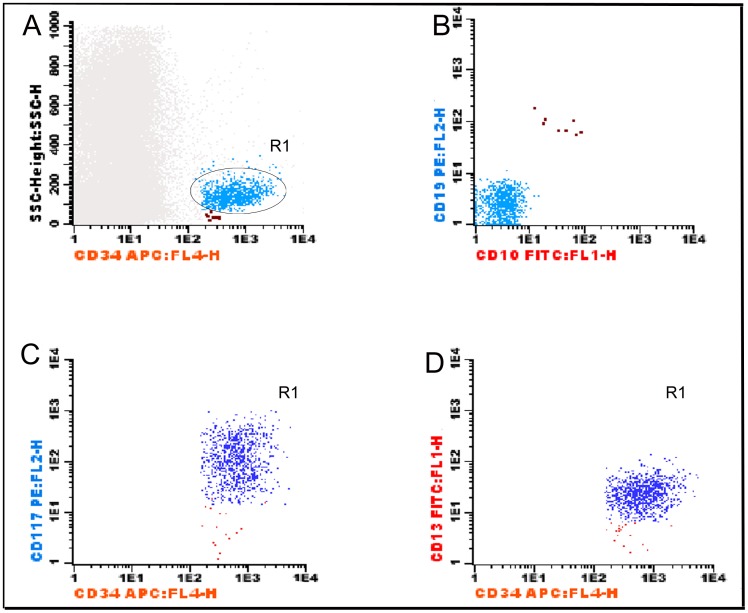
analysis of the subsets of CD34^+^ cells. (combination CD19/CD10/CD45/CD34 in A and B and CD13/CD117/CD45/CD34 in C and D). In a first step, (A) all CD34^+^ cells were selected in the SSC/CD34 plot. Two populations can be observed: SSC^low^ (in brown) that are CD19^+^/CD10^+^ (B-cell precursors) on the CD19/CD10 plot (B), and SSC^int^/CD34^+^/CD19^−^ (light blue). These cells are characterized as myeloid subsets of CD34^+^ cells (C and D) when analysed in the CD13/CD117/CD45/CD34 combination. Among SSC^int^/CD34^+^ cells, the CD34^+^/CD117^+^ subset (dark blue) and CD34+/CD117^−^ subset (red) were defined by their expression of CD117 and CD13. Myeloblasts SSC^int^/CD34^+^/CD117^+^/CD13^+^ (dark blue) and immature cells SSC^int^/CD34^+^/CD117^−/^CD13^−^ (red).

CD34^+^ cells (6 abnormalities): increase of CD34^+^ cells, decrease of B-cell precursors (CD34^+^/CD19^+^/CD10^+^), increase of myeloblasts (SSC^int^/CD13^+^/CD117^+^/CD34^+^) (Fig. 3), increase of immature non-lymphoid precursors (SSC^int^/CD34^+^/CD117^−^ or SSC^int^/CD34^+^/CD13^−^), and abnormal expression of CD7 or CD56 in CD34^+^ cells.

Immediately after staining, samples were acquired in a FACSCalibur flow cytometer (Becton Dickinson – BD Biosciences, San José, CA, USA) using the CellQuest software (BD Biosciences). Information of least 100,000 events was acquired for each sample. Data analysis was made using the Infinicyt software (Cytognos, Salamanca, Spain). We computed the total number of granulocytic and monocytic abnormalities and those of CD34^+^ cells. The sum of all these abnormalities was called “total alterations” Concerning CD34^+^ cells, we calculated the total percentage of positive cells among all nucleated cells, and the subsets: B-precursor cells (CD34^+^/CD19^+^/CD10^+^), SSC^int^/CD34^+^/CD13^+^ and SSC^int^/CD34^+^/CD13^−^ cells [17], [20], [23].

### Statistics

Differences among groups and relations between phenotypic features and other risk factors were analyzed by non-parametric tests (Mann-Whitney, Kruskall-Wallis and Spearman’s test). Overall survival of the patients according to WPSS, IPSS and IPSS-R was calculated by the Kaplan-Meier method followed by the log-rank test. Cluster analysis for cytometric data was performed according to the Ward algorithm [25]. We tested the WHO classification, WPSS, IPSS and IPSS-R as categorical variables and all other variables entered the models as continuous variables. Patients were censored if they underwent chemotherapy or bone marrow transplantation.

The prognostic relevance of PB counts, WHO classification, the three prognostic scores analyzed, revised cytogenetic categories, % BM blasts, total CD34^+^ cells, SSC^int^/CD34^+^/CD13^+^ and SSC^int^/CD34^+^/CD13^−^ cells, as well as the “total alterations” was analyzed by univariate and multivariate Cox regressions. The limits for input were p = 0.05 and p = 0.1 for output. Backward conditional step-wise selection was applied to all calculations with the exception of the multivariate comparison of the three risk stratification scores, where the forward stepwise conditional mode was used.

Since our aim was to examine if variables derived from flow cytometry analysis which had impact on patients’ survival could add prognostic information to the three scores analyzed we created three multivariate models, testing always the variables with p<0.05 in the univariate Cox models together with one of the three risk classifications systems. Furthermore, we run the three risk stratification systems together in a multivariate proportional hazard model.

The internal stability of the models was tested by bootstrap resampling [26]–[39]. In brief, new data sets with the same size of the original one were created by random sampling with replacement. In each new bootstrap data set, a patient may be represented once, several times or not at all. Cox regressions with the same conditions as in the original data set were performed for each of these new bootstrap data sets.

The 95% confidence intervals of the R-squared values were calculated based on the values obtained by bootstrapping. In each bootstrap set we analyzed the consistency of the three classifications, i.e. whether with increasing risk group the hazard ratio increased. SPSS 8.0 (to calculate the R values), SPSS 15.0 and Winstat softwares were used for calculations.

## Results

A total of 101 patients (64 males and 37 females) were included in this prospective study. The median age at diagnosis was 64 years (15–93). Among the WHO types, the majority of the cases had refractory cytopenias with multilineage dysplasias (RCMD) and had a good risk cytogenetics (Table 2). Concerning the IPSS, most patients were classified as low or intermediate-1 risk.

**Table 2:** Clinical features, PB counts and prognostic scores of the patients.

BM Blasts (%)	0–2% N = 54	2%–5% N = 19	5%–10% N = 13	>10% N = 15
Age (years) Median	63	62	70	66
Min – Máx	15–93	28–84	53–78	40–79
Sex: Men/Women	32/22	14/15	8/5	10/5
**Hemoglobin (g/dL)**				
Median	9.1	8.1	9.1	8.9
Mìn – Máx.	5.0–15.1	4.8–16.2	6.6–13.5	5.1–12.6
**Neutrophils x10^9^/L**				
Median	1.85	1.30	1.20	1.0
Mín. – Máx	0.20–5.70	0.14–4.70	0.40–4.50	0.40–4.30
**Platelets (x10^9^/L)**				
Median	181.0	108.0	51.0	78.0
Min – Máx	5.0–854.0	18.0–456.0	16.0–139.0	16.0–841.0
**WHO type**				
RA	7	0	0	0
RARS	0	0	0	0
RCMD	31	16	0	0
RCMD-SA	14	3	0	0
RAEB-1	0	0	11	0
RAEB-2	0	0	2	15
5Q – Syndrome	2	0	0	0
**WPSS**				
very low risk	3	0	0	0
low	22	6	0	0
intermediate	20	11	5	0
high risk	4	2	7	9
very high risk	0	0	1	5
**Revised cytogenetic risk**				
very low	1	0	0	0
good	42	17	12	6
intermediate	4	3	0	3
high/very high	0	0	0	1/7
**IPSS**				
low risk	25	7	0	0
intermediate-I	22	10	11	0
intermediate II	3	2	2	7
high risk	0	0	0	7
**IPSS-R**				
very low risk	12	0	0	0
low risk	29	7	1	0
intermediate	5	10	6	2
high risk	3	2	5	6
very high risk	0	0	1	6

Comparing the cytogenetic risk classification used in IPSS and the revised one, only 17 cases changed category. The change in risk categories after reclassification of the patients according to IPSS-R is shown in Table 3. The patients with “IPSS low risk” were divided into “IPSS-R very low” and “low risk”, but those with “IPSS intermediate risk” were distributed into many categories in IPSS-R, while the majority of those with “IPSS high risk” were classified as “IPSS-R very high risk”

**Table 3:** Changes in risk category of the patients after reclassification by IPSS-R.

	IPSS low	IPSS int I	IPSS int II	IPSS high
IPSS-R very low	14	0	0	0
IPSS-R low	18	21	1	0
IPSS-R intermediate	1	17	5	0
IPSS-R high	0	8	7	2
IPSS-R very high	0	0	1	6

Concerning immunophenotypic abnormalities, all cases presented at least 2 alterations **(** Table 4). Cross-lineage aberrant expressions (CD7 and CD56) in myeloid CD34^+^ cells were found in 54% of the cases. Percentage of BM blasts was correlated with total CD34^+^ cells (r = 0.66; p<0.0001); “CD34^+^/CD13^+^”, and “CD34^+^/CD13^−^ cells” (r = 0.51; p<0.0001 and r = 0.42; p<0.0001 respectively).

**Table 4:** distribution of the phenotypic abnormalities examined according to the median percentage of BM blasts in cytology.

% blasts	0–2	2.5–5	5.5–10	>10
Total CD34^+^	0.66	1.95	2.37	7.1
CD34^+/^CD13^+^	0.28	0.78	1.35	5.53
CD34^+/^CD13^−^	0.23	0.39	0.50	0.96
CD34^+/^CD19^+^	0 (0–0.72)	0 (0–0.29)	0 (0–0.3)	0 (0–0.17)
Nr. of abnormalitiesin CD34^+^ cells	1 (0–5)	3 (0–6)	4 (1–5)	4 (1–6)
Total number of alterations	5 (2–10)	6 (2–12)	8 (3–10)	7 (3–13)

The total number of phenotypic abnormalities increased with the increasing risk in the classifications: WHO type (r = 0.27; p = 0.005), WPSS (r = 0.40; p<0.0001), IPSS (r = 0.36; p<0.0001) and IPSS-R (r = 0.49; p<0.0001). The total number of phenotypic abnormalities was negatively correlated with the hemoglobin levels (r = - 0.40; p<0.0001), but there were no significant correlations with PB neutrophil and platelet counts.

### Survival Analysis

**Figure 4:**
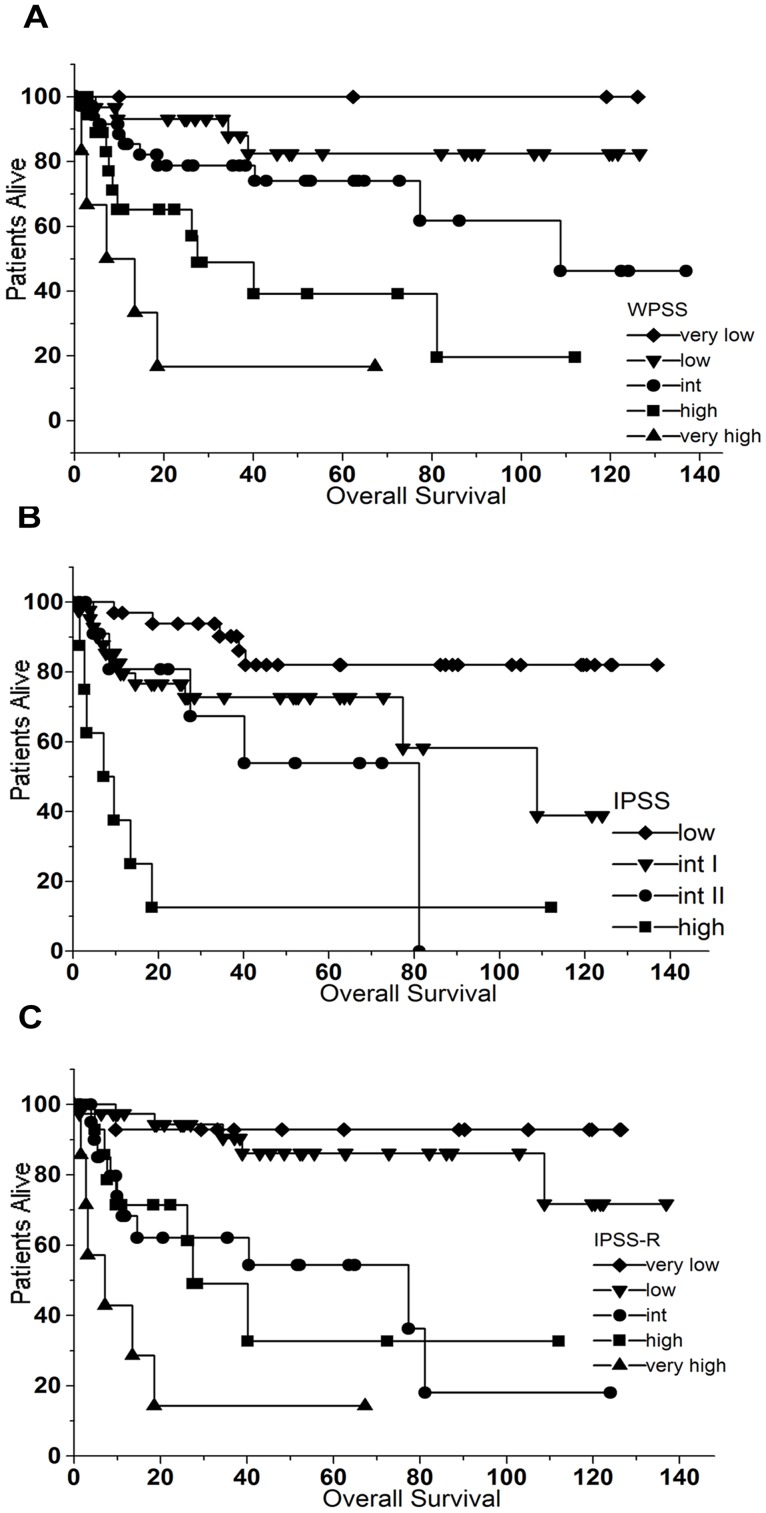
Kaplan-Meier estimates for overall survival of the patients according to WPSS (A), IPSS (B) and IPSS-R (C). All three scores were able to separate patients with a significantly different survival (p<0.0001).

Median duration of the patients’ follow-up was 28 months (1–134). At the end of the observation period 29 patients had died. IPSS, IPSS-R and WPSS were able to stratify patients with a different survival (p<0.0001 for all three scores) (Fig. 4).

In the univariate Cox analysis (Table 5) all the classification systems and prognostic scores analyzed had a significant association with the overall survival of the patients.

**Table 5:** Univariate Cox analysis of the three prognostic scores examined with 95%CI intervals for R^2^ calculated by boostrap resampling (in brackets).

	HR	R^2^	p
**IPSS**		0.066 (0.012–0.166)	<0.0001
Low risk	0.068 (0.0214–0.220)		
Intermediate I	0.207 (0.0803–0.532)		
Intermediate II	0.304 (0.0947–0.978)		
High risk	1		
**IPSS-R**		0.080 (0.0106–0.110)	<0.0001
Very low risk	0.0238 (0.0028–0.250)		
Low risk	0.0553 (0.0163–0.188)		
Intermediate	0.2891 (0.1033–0.809)		
High risk	0.3115 (0.135–0.0.938)		
Very high risk	1		
**WPSS**		0.050 (0.004–0.126)	<0.0001
Very low risk	0.00005 (0–0.0001)		
Low risk	0.064 (0.017–0.248)		
Intermediate	0.165 (0.056–0.499)		
High risk	0.416 (0.141–1.23)		
Very high risk	1		

Age was not related to survival but diminished hemoglobin values, revised cytogenetic risk categories and the percentage of BM blasts were risk factors. The same was true for the total number of immunophenotypic abnormalities found, number of abnormalities in monocytes and CD34^+^ cells as well as total number of CD34^+^ cells and their myeloid subsets (SSC^int^/CD34^+^/CD13^+^ and SSC^int^/CD34^+^/CD13^−^) (Table 6).

**Table 6:** Prognostic single variables that showed a significant association with overall survival in the univariate Cox analysis.

	B	HR	R^2^	p
Hemoglobin level	−0.271	0.763	0.03441	0.001
Revised cytogenetic classification	0.544	1.731	0.03136	0.002
% BM blast in cytology	0.134	1.144	0.07828	<0.0001
Total % CD34^+^ cells	0.182	1.200	0.09985	<0.0001
%CD34^+^/CD13^+^ cells	0.256	1.292	0.08809	<0.0001
%CD34^+^/CD13^−^ cells	0.343	1.409	0.02709	0.003
Number of monocytic abnormalities	0.427	1.534	0.01361	0.022
Number of CD34^+^ abnormalities	0.336	1.400	0.03497	<0.0001
Total number of immunophenotypic abnormalities	0.265	1.303	0.05924	0.0001

In the multivariate analysis, comparing the three prognostic scores studied, only IPSS-R remained as an independent risk factor of overall survival (p<0.0001; R^2^ = 0.07952). In the bootstrap resampling, IPSS-R was present in 88% of the new models, IPSS in appeared in 20% and WPSS in 18% of the new data sets. The variation of the R-squared values in the bootstrap resampling sets was: median 0.0462 (CI 95%: 0.0148–0.0960) for IPSS-R, median 0.0433 (CI 95%: 0.0106–0.1179) for IPSS and median 0.06993 (CI 95%: 0.0139–0.1156) for WPSS.

In the univariate analysis, the risk stratifications were consistent (B values continuously increasing with increasing risk) in 84% of the bootstrap sets analyzed with the IPSS or WPSS score and in 77% of the sets analyzed with the IPSS-R score.

When joining together WPSS and phenotypic variables (“CD34^+^/CD13^+^ cells”, “CD34^+^/CD13^−^ cells” and “total alterations”), only WPSS and “CD34^+^/CD13^+^ cells” remained in the final multivariate Cox model (p = 0.02 and p = 0.004 respectively) (Table 7). In the bootstrap resampling stability test, WPSS remained in 88%, “CD34^+^/CD13^+^ cells” in 79%, “total alterations” in 44% while “CD34^+^/CD13^−^ cells” remained only in 5% of the models.

**Table 7:** Results of the multivariate analysis considering each of the scores analyzed together with the phenotypic variables presenting an impact on overall survival of the patients.

Risk stratification score	Prognostic indexHR 95% CI	R^2^ score	CD34^+^/CD13^+^ cellsHR 95% CI	R^2^ CD34^+^/CD13^+^ cells	Total alterationsHR 95% CI	R^2^ Total alterations
Model 1*						
**WPSS**						
very low risk	5.22×10^−7^		1.135			
low risk	0.127 (0.027–0.59)	0.00212	(1.040–1.241)	0.0253	−. −	−. −
intermediate	0.294 (0.080–1.073)					
high risk	0.564 (0.169–1.878)					
very high risk	1					
Model 2**						
**IPSS**			1.162	0.0433	1.216	0.0193
			(1.068–1.264)		(1.047–1.412)	
Model 3***						
**IPSS–R**						
very low	0.040 (0.004–0.373)		1.118			
low risk	0.094 (0.024–0.369)	0.0331	(1.024–1.220)	0.0179	−. −	−. −
intermediate	0.408 (0.129–1.284)					
high risk	0.356 (0.111–1.139)					
very high risk	1					

In the multivariate analysis of IPSS and the three immunophenotypic variables considered (Table 7), only “CD34^+^/CD13^+^ cells”, and “total alterations” remained in the model (p = 0.0005 and p = 0.01 respectively), whereas the IPSS score was eliminated. In the stability test, IPSS appeared in 69% “CD34^+^/CD13^+^ cells” in 79%, “total alterations” in 55% and “CD34^+^/CD13^−^ cells” in only 3% of the models.

Finally, in a multivariate analysis containing IPSS-R and flow cytometric variables, only IPSS-R and “CD34^+^/CD13^+^ cells” entered the model (p = 0.0012 and p = 0.01 respectively). In the bootstrap resampling, IPSS-R was found in 96%, “CD34^+^/CD13^+^ cells” in 76%, “total alterations” in 17% and “CD34^+^/CD13^−^ cells” in only 5% of the models.

**Figure 5:**
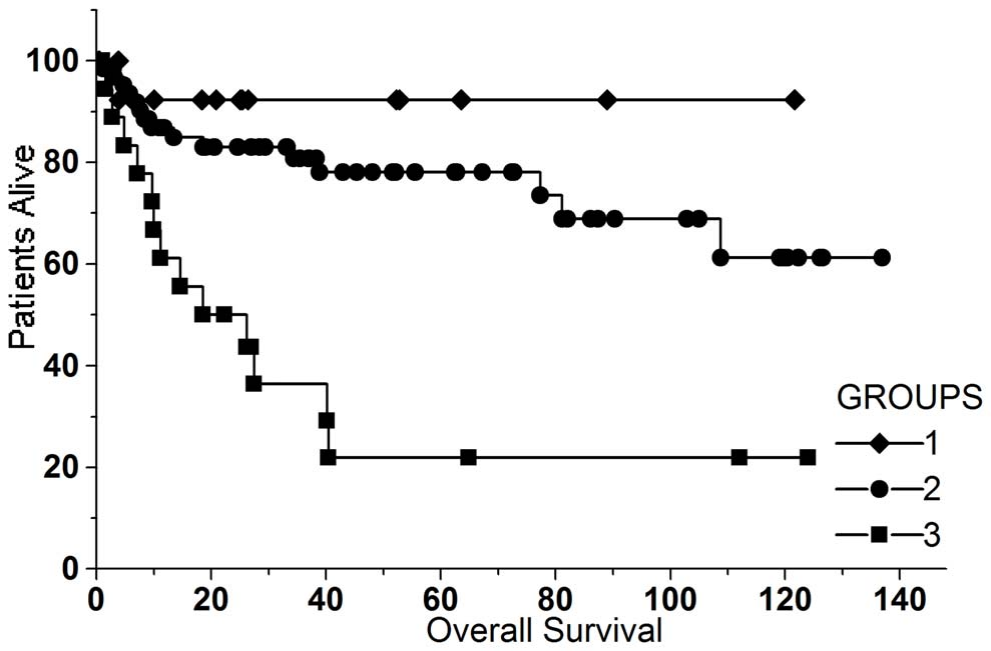
overall survival of the patients according to the number of CD34^+^/CD13^+^ cells separated in three groups with a different survival by cluster analysis. Group 1 median: 0.07 (0.02–0.11), group 2 median: 0.46 (0.14–2.36) and group 3: median 5.07 (2.90–27.9) (p<0.0001).

After normalization by logarithmic transformation, the variable “CD34^+^/CD13^+^ cells” was submitted to a cluster analysis with the Ward algorithm, which suggested 3 groups: group 1 (16 cases) median CD34^+^/CD13^+^ cells = 0.07 (0.02–0.11); group 2 (69 cases) median CD34^+^/CD13^+^ cells = 0.46 (0.14–2.36) and group 3 (15 cases) median CD34^+^/CD13^+^ cells = 5.07 (2.9–27.9). These groups had a significantly different survival in the log-rank test (p<0.0001) (Fig. 5).

## Discussion

Myelodysplastic syndromes comprise a heterogeneous group of clonal hematopoietic disorders concerning patients’ clinical behavior and laboratory features [4]–[10]. In recent years, important advances in the understanding of its pathophysiology have been achieved and some disease-altering treatment strategies have been established [11], [12], [40]–[43]. In this context, new prognostic factors may enable us to improve the choice of the best therapeutic options.

In the present work, which is prospective and mono-institutional, from an Institution not participating in the International Working Group for Prognosis in MDS (IWG-PM) project [9] we compared the prognostic value of two well-established risk scores (IPSS and WPSS) and the newly described revised IPSS (IPSS-R). Furthermore, we examined if variables obtained by immunophenotyping could improve the risk stratification obtained by each of the clinical score systems.

IPSS is based on cytogenetic risk stratification, number of PB cytopenias and a rough categorization of BM blasts. These features have long been recognized as independent risk factors in MDS. WPSS incorporated the WHO classification of MDS that is also, per se, able to separate groups of patients with a different outcome [5], [10]. It also includes the concept that anemia and transfusion dependency are an important hallmark of disease severity. Anemia is the most common peripheral cytopenia in MDS and is associated with the most important morbidity such as transfusion dependency and iron overload [4], [10]. This was also observed in our patients, where the hemoglobin level was inversely related to survival, but neutrophil and platelet counts were not. This is also in keeping with the findings of the IWG-PM [9], and with another previous study of our group [5].

The recent IPSS-R classification system is not only based on a more refined cytogenetic risk classification, but also on a more detailed splitting of BM blast count and PB values. In the present work, we could validate this new score system in a patient cohort which had not participated in the IWG-PM study.

Concerning the internal validation of our model, different solutions could be chosen. An excellent method is data splitting, where a sub-sample will serve for model selection and the remaining cases will be used for the internal validation. But in our study, the relatively low number of cases in some subgroups precluded the use of this methodology. Alternative methods would be the jack-knife procedure [29]–[35], or the bootstrap resampling, which can be considered an elaboration of the jack-knife analysis. Bootstrap resampling is interesting, since it allows to investigate the consistency of the inclusion of each variable in the regression model [29]–[39] and to estimate “internal validity”, which means that the model closely mirrors the survival patterns of the original data [38]. Before a model is applied in routine clinical practice, it should however, be tested for external validity, which implies testing its generalizability with other groups of patients, preferentially in other Institutions.

In linear regressions, R^2^ helps to estimate the explanatory power of a model [33], [36], [37]. In a similar way, R-squared values can be calculated for survival regressions, where they asses the proportion of variability in the patients’ outcome explained by each variable. But here, the R-squared values decrease with increasing percentage of censored patients and earlier censoring. Moreover, the generalized R-squared does not represent exactly the proportion of variance of the dependent variable explained by the independent variable, but provides rather an estimate of association between these variables [38]. Therefore, the R-squared value may be small even in good fitting models, and is able to summarize considerable information.

When comparing the three classifications in the bootstrap sets in the univariate analyses, we found that the median R-squared values for IPSS and IPSS-R were rather similar, but the variation among the 100 new sets was larger for IPSS. In the multivariate analysis joining together the three scores, IPSS-R was present in most new models but the two other scores were present in only a small number of the bootstrap sets. Besides, when joining together each score with the same immunophenotypic variables in multivariate analyses, the IPSS score entered only in 69% of the bootstrap sets, whereas IPSS-R in 96%. For all these reasons the IPSS-R score may be regarded as a more consolidated risk classification system. This may be due to the fact that in IPSS-R the degree of the peripheral cytopenias and the percentage of bone marrow blasts are stratified more in detail.

A head-to-head comparison among the three scores, as done in our study has not been reported so far, to the best of our knowledge. So, a confirmation of our results in larger cohorts of patients should be performed.

Immunophenotyping is now a well-established ancillary technique for the diagnosis of MDS [13]–[18] and has been standardized by the European LeukemiaNet [15], [23]. This technology is able to demonstrate, in a simple and rapid way, the degree of functional damage of the abnormal clone, revealing maturational abnormalities in the myelomonocytic [14], [15]–[18], [22], [23] and erythroid lineages [14], [15] through abnormal antigen expression, asynchronous antigen co-expression as well as cross-lineage aberrant expressions. Moreover, increase in number of CD34^+^ progenitors, as well as their phenotypic abnormalities corresponding to leukemia-associated phenotypes (LAIPs), are also frequently found [15], [22], [23]. Most consensus reports recommend the analysis of the myelomonocytic lineages. However, the analysis of the erythroid precursors is not mandatory, because only few antibodies are available for this lineage. Besides, sample preparation may lyse a part of the erythroblasts, which can affect the measures obtained [13], [15], [18], [23].

Accordingly, several publications have emphasized the prognostic relevance of some of the features obtained, such as the total number of CD34^+^ cells and their subsets corresponding to early myeloid progenitors, aberrant expressions of CD34^+^ cells as well as the total number of phenotypic abnormalities detected [13]–[23]. An increase of the latter has been observed during disease progression [18], [20], [44], [45], [47] and has been related to a worse outcome after BM transplantation [47]. Some study groups reported a relation between the total number of phenotypic abnormalities and IPSS [14], [18], [43], [46] or WPSS [17], [20]–[22]. Aberrant cross-lineage antigen expressions in CD34^+^ cells, may be found in up to 38% of the cases [22], [45], can be detected in some patients with a low BM blast count and have been associated with disease progression, transfusion dependency and a shorter survival. Several flow scores have also been described, but there is no agreement about their use in clinical practice [18], [22], [43].

For these reasons, our main question was whether flow cytometric features could refine the prognostic value not only of IPSS-R, but also IPSS and WPSS. This comparison has not yet been done to the best of our knowledge. In our study, the number of phenotypic abnormalities found in monocytes and in CD34^+^ cells but not those of maturing granulocytes presented a significant relation to the patients’ survival in the univariate analysis. Consequently, also the total number of abnormalities detected was related to the overall survival of the patients, but had a lower relevance. The total number of CD34^+^ cells and their myeloid subsets were the most important features capable to predict the patients’ outcome. So we decided to examine in multivariate analyses if one of these variables could retain an independent prognostic value together with IPSS, WPSS or IPSS-R. We chose to test “CD34^+^/CD13^+^ cells”, “CD34^+^/CD13^−^ cells” and “total alterations” as these features are easy to obtain and standardize and can be measured in all cases, including those lacking cytogenetic information or having a normal karyotype.

In the multivariate analysis comparing all scores examined with flow cytometric features, the number of SSC^int^/CD34^+^/CD13^+^ cells was the strongest independent feature that could add prognostic information to the clinical scores examined. These cells represent the major fraction of CD34^+^ cells and correspond to an objective measure of BM myeloid blasts [17], [20], [42], [48], [49]. They may be quantified in all patients, differently from LAIPs that are present in only about half of the cases, as was also observed in our patients [18], [23], [45].

Although all clinical scores consider the percentage of BM blasts in a categorized way or indirectly in the WHO types, our results demonstrate that enumeration of myeloid progenitors by FCM is able to increase risk stratification of the patients.

Different reasons may explain this phenomenon. First, we have to remind that every form of stratification, as done for the BM blast numbers in the score systems, will diminish considerably the test power [50]–[52]. Therefore, a part of the information “lost” by categorizing the blast numbers will be regained by adding the continuous flow cytometric variables in the Cox regression.

Moreover, blasts are counted by the human observer under the microscope [41]. This kind of measure is subject to considerable inter-observer variability and “expert opinion”. Besides, a relatively low number of blasts are counted in the specimens. So, the “manual” blast count cannot be regarded as a very precise measure (a maximum of total 500 cells are counted in routine work), especially when compared with flow cytometric data which are based on a large number of cells (usually at least 100000 events) [53]. Moreover, especially in MDS cases, the reproducibility of cell typing is low, due to the high degree of cell atypias which hampers considerably cell classification [41], [53]. Both kinds of samples can have varying degrees of hemodilution when compared to BM histological specimens [23], [54] resulting in different counts when assessed by both techniques. For all the reasons above one can consider both features as related but independent measures [23].

In order to illustrate the importance of the CD34^+^/CD13^+^ cell counts we performed a cluster analysis and stratified the data into three non-overlapping groups of patients with a different survival. These groups separated mainly patients with a low number of SSC^int^/CD34+/CD13^+^ cells and a low blast count in cytology, emphasizing again the importance of considering subtle abnormalities in the hematopoietic progenitors for the evaluation of the aggressiveness of the abnormal clone.

It was interesting to note that comparing IPSS with flow cytometric features, these last ones were more powerful in predicting patients’ outcome. This may be due to the fact that IPSS considers only the number of PB cytopenias and not the degree of BM failure. Besides, the categorization of BM blasts assigns more points to counts above 10%. However, in our cluster analysis we could separate three groups with a different survival mainly among cases with low numbers of SSC^int^/CD34^+^/CD13^+^ cells and therefore immunophenotypic variables were able to add important prognostic information to IPSS. This underlines the importance to refine stratification of patients with a low BM blast count in cytology, which was made in the revised IPSS-R, where more points are assigned also low BM blast counts.

Comparing WPSS with immunophenotypic variables, the clinical score remained in the model together with “SSC^int^/CD34^+^/CD13^+^ cells”, which were stable in the bootstrap resampling, although “total alterations” entered a large number of new data sets. However, in the comparison using IPSS-R, the best clinical score in our study, only “SSC^int^/CD34^+^/CD13^+^ cells” remained as a stable independent risk factor. However, all these results should be validated further in a larger cohort of patients.

We could confirm previous reports that maturation abnormalities in the granulocytic series and decrease in B-cell precursors are rather an adjuvant criterion for the differential diagnosis of MDS with a normal karyotype and non-clonal cytopenias, but have a limited impact on patients’ survival [13], [18], [20], [55], [56]. Interestingly, the number of abnormalities and especially the percentage of monocytes were significant risk factors, at least in the univariate analysis. It has been shown that, with the progression to AML, the number of mature monocytes decrease [14], [16], [20].

In conclusion, our data corroborate that FCM is able to add important prognostic information to all the prognostic scores studied. It is feasible in all patients, is especially useful in cases with a normal karyotype and gives more detailed information about the maturation abnormalities and the number of myeloblasts. Immunophenotyping is complementary, and should be used together with the clinical scores for more precise risk stratification of MDS patients.
